# Threat of establishment of non-indigenous potato blackleg and tuber soft rot pathogens in Great Britain under climate change

**DOI:** 10.1371/journal.pone.0205711

**Published:** 2018-10-12

**Authors:** Peter Skelsey, Sonia N. Humphris, Emma J. Campbell, Ian K. Toth

**Affiliations:** 1 Information & Computational Sciences, James Hutton Institute, Dundee, United Kingdom; 2 Cell & Molecular Sciences, James Hutton Institute, Dundee, United Kingdom; College of Agricultural Sciences, UNITED STATES

## Abstract

Potato blackleg and soft rot caused by *Pectobacterium* and *Dickeya* species are among the most significant bacterial diseases affecting potato production globally. In this study we estimate the impact of future temperatures on establishment of non-indigenous but confirmed *Pectobacterium* and *Dickeya* species in Great Britain (GB). The calculations are based on probabilistic climate change data and a model fitted to disease severity data from a controlled environment tuber assay with the dominant potato blackleg and soft rot-causing species in GB (*P*. *atrosepticum*), and three of the main causative agents in Europe (*P*. *carotovorum* subsp. *brasiliense*, *P*. *parmentieri*, *Dickeya solani*). Our aim was to investigate if the European strains could become stronger competitors in the GB potato ecosystem as the climate warms, on the basis of their aggressiveness in tubers at different temperatures. Principally, we found that the tissue macerating capacity of all four pathogens will increase in GB under all emissions scenarios. The predominant *Pectobacterium* and *Dickeya* species in Europe are able to cause disease in tubers under field conditions currently seen in GB but are not expected to become widely established in the future, at least on the basis of their aggressiveness in tubers relative to *P*. *atrosepticum* under GB conditions. Our key take-home messages are that the GB potato industry is well positioned to continue to thrive via current best management practices and continued reinforcement of existing legislation.

## Introduction

Potato blackleg and soft rot, caused by *Pectobacterium* and *Dickeya* species, are among the most significant bacterial diseases affecting potato production globally [1–5]. In Great Britain (GB), these diseases are caused predominantly by *Pectobacterium* species, with the vast majority of cases being *P*. *atrosepticum* (Pba) [6–8]. Blackleg and soft rot are longstanding problems in GB, as in many other potato growing regions of Europe and elsewhere, and although incidence has been significantly reduced since the 1960s, thanks largely to improvements in storage and seed certification, recent years have seen a marked upward trend in incidence in seed potato crops [6, 7]. There has also been an increase in blackleg and soft rot incidence in continental Europe over the last ten years, corresponding with an increase in the number of causative organisms including *P*. *carotovorum* subsp. *brasiliense* (Pbr), *P*. *parmentieri* (Ppa), and *D*. *solani* (Dsol) [5]. Since it was first isolated in Europe in 2004, Dsol has spread rapidly across the continent and was, until recently, the predominant pathogen responsible for blackleg and soft rot incidences [5, 9, 10]. More recently, both *P*. *carotovorum* subsp. *brasiliense* (Pbr) and *P*. *parmentieri* (Ppa) have also been responsible for considerable disease incidence [11, 12].

*Pectobacterium* and *Dickeya* species cannot survive in the soil between potato crops in a crop rotation system in GB, and it is generally accepted that the major source of field inoculum is the latently infected seed (mother) tubers [13, 14]. When the ‘mother’ tuber rots, the bacteria are released into the soil and are transmitted by soil water to contaminate neighboring progeny tubers. Czajkowski et al. [15] showed that the bacteria in soil can also colonize potato roots and subsequently move via the vascular system into progeny tubers. When progeny tubers are infected, field symptoms include reduced emergence, wilting, chlorosis, tuber and stem rot, blackleg, haulm (stalk) desiccation and plant death [16]. If environmental conditions are not suitable for disease development the bacteria can survive in latent form, which can lead to extensive soft rot in tubers in storage. Most importantly, latently infected seed (mother) tubers may form the next generation of seed, resulting in carryover of inoculum to the following field generations.

Temperature plays a critical role in mother-tuber rotting; it has been demonstrated that *Pectobacterium* spp. grow better and are more pathogenic at lower temperatures (<25°C) compared to *Dickeya* spp. (>25°C) [2, 3, 9, 17–20]. Temperature has been found to determine species presence and the expression of pathogenicity factors [21–23], with numerous studies demonstrating the effect of temperature on species selection [3, 19, 24–27]. If more than one species is present inside a rotting mother tuber, it has been shown that temperature modulates which pathogen will predominate [28]. A species exposed to its optimal temperature range will therefore have the advantage of maximum growth rate allowing it to reach critical numbers ahead of other competing species, both within-individual and between-individual mother tubers in the field [17]. Another important environmental factor for mother-tuber rotting is soil water level. Presence of a water film on the tuber surface induces development of anaerobic conditions in the mother tubers, thereby favoring bacterial multiplication and initiation of rotting [29].

The predominant *Pectobacterium* and *Dickeya* strains in Europe (Ppa, Pbr, and Dsol) have been confirmed in GB but have yet to become established [6]. As the trend towards hotter summers in GB in recent decades is expected to continue [30], there is a concern that they could cause increased disease problems in the future, given the known effects of temperature on aggressiveness, pathogenicity differentiation, competition and species selection. There is therefore a need to assess the potential for a temperature-induced shift in the prevalence of non-indigenous but confirmed *Pectobacterium* and *Dickeya* spp. in GB, to guide strategies for agricultural adaptation to climate change. An increased frequency of drier summers is also expected for GB, which could affect soil moisture levels and thus risk of mother-tuber rotting. Projections of the relationship between land suitability for potato production and water resource availability show that growing rainfed potatoes in GB will become increasingly difficult as the climate changes, with a consequent increase in demand for supplemental irrigation [30, 31]. It is anticipated that this demand will be met in the short-term via additional water abstraction licenses (e.g. abstraction in winter, and storage in a reservoir) and license trading, and through various short-term coping strategies, such as improved irrigation technology (scheduling) and equipment, modification of soil structure to improve soil moisture retention, and installation of rainwater harvesting equipment. In the long-term, however, a geographical shift of potato production to regions with suitable agroclimate and water availability is considered as the most viable option for the GB potato industry [30, 31]. Thus, a further research priority is to assess the implications of such a geographical shift on risk of establishment of the non-indigenous European *Pectobacterium* and *Dickeya* strains.

For the purposes of a preliminary climate change risk assessment for non-indigenous *Pectobacterium* and *Dickeya* species in GB, we focus on the effect of temperature on tuber macerating capacity, given the critical role of mother-tuber rotting to the epidemiology of these pathogens. We also evaluate agronomic scenarios for relocation of GB potato production from the semi-arid east to areas of higher rainfall on the west. Our goals were to: (1) quantify the effect of different temperatures on the aggressiveness of Pba, Pbr, Ppa, and Dsol in potato tubers; (2) investigate if the non-indigenous European strains and species (Pbr, Ppa, Dsol) could become stronger competitors in the GB potato ecosystem under future climates, on the basis of their rate of development within (mother) tubers relative to Pba; and (3) compare the effect of temperature regimes in current crop locations and areas of higher rainfall on the relative aggressiveness of Pba, Pbr, Ppa, and Dsol in potato tubers. We used a newly developed empirical model fitted to disease severity data from a controlled environment tuber assay, and a Bayesian modelling framework together with GB potato crop distribution data to project future maceration ability under various CO_2_ emissions and agronomic scenarios.

## Materials and methods

### Tuber assay

A tuber slice assay was carried out to assess the macerating capacity of *Pectobacterium atrosepticum* (Pba), *P*. *carotovorum* subsp. *brasiliense* (Pbr), *P*. *parmentieri* (Ppa), and *Dickeya solani* (Dsol) isolates at five different temperatures: 18°C, 21°C, 25°C, 28°C and 32°C. Tuber slice assays are routinely used for measuring disease severity (e.g. [32, 33]), as the number of replicates required for a whole tuber assay is often impractical in a laboratory environment. We used four isolates each for Pba and Dsol, and two isolates each for Pbr and Ppa. Bacterial strains were grown overnight, washed and resuspended into 10 mM MgSO_4_ to a final concentration of 10^7^ CFU/ml_._ Maris piper tubers were surfaced sterilized for 10 mins in 5% sodium hypochlorite, rinsed in distilled water and cut into 7mm thick slices using a sterile knife. A core was removed from the center of each slice to make a well 5mm deep and 7mm in diameter. The slices were inoculated by pipetting 50μl of the 10^7^ CFU/ml bacterial suspension into each well [34]. Three potato slices obtained from 3 different potato tubers was used for each bacterium tested and as a control, slices were inoculated with 50μl of 10 mM MgSO_4_. The tuber slices were placed on petri dishes and positioned randomly on wet tissue paper in a sealed box and incubated for 72 hours at the 5 temperatures. Disease severity was expressed as the mean diameter of the soft-rotted tissue area (lesion length). The entire experiment was independently replicated two times, using different cultures for inoculum in each experiment.

### Temperature-dependent model for tuber maceration

A model to describe the effects of temperature on disease severity (lesion size) in tubers was developed by fitting the simplified temperature response function of Yin et al. [35, 36] to the lesion size data at the third day after inoculation. The function uses a pathogen’s cardinal temperatures to estimate the shape parameter and the temperature response:

$$r=L_{\max}\left(\frac{T_{\max}-T}{T_{\max}-T_{\mathrm{opt}}}\right){\left(\frac{T}{T_{\mathrm{opt}}}\right)}^{\frac{T_{\mathrm{opt}}}{T_{\max}-T_{\mathrm{opt}}}}$$ (1)

if *T*_min_ ≤ *T* ≤ *T*_max_ and 0 otherwise, where *r* is lesion size, *L*_max_ is maximum lesion size, *T* = mean temperature (°C) during maceration, *T*_max_ = maximum temperature for maceration, and *T*_opt_ = optimum temperature for maceration. The advantages of the Yin function (a beta function) compared with other temperature response functions include that it has only three parameters, and each has a clear biological meaning [35]. The function gives a smooth curve as opposed to a series of lines with abrupt changes between them. The function combines the advantages of several equations: an exponential response at low temperatures, a positive linear response at intermediate temperatures, a parabola response at optimum temperatures, and a negative response at high temperatures. The model has been validated with data sets of crop growth (e.g. [35, 37]) and various plant-pathogen interactions (e.g. [38–41]). For comparative purposes we also fitted a linear model, with lesion size as the response variable and temperature as the independent variable. The linear model is often convenient and effective when the temperature does not approach or exceed the optimum, which could be the case in one or more of the assays.

Tuber slice and experimental replicates were treated as independent (*n* = 6 host-pathogen interactions per temperature). The models were fitted separately to the data for Pba, Pbr, Ppa, and Dsol using a Bayesian framework that produces distributions of model parameter values and residual error that provide a complete characterization of model uncertainty that can be seamlessly propagated through to the climate change risk assessment. We used Markov Chain Monte Carlo (MCMC) methods via Gibbs sampling with the software WinBUGS 1.4. The basic idea of the MCMC method, and in particular of the Gibbs sampling algorithm, is that the program steps through the unknown parameters one at a time, estimating the posterior distribution of each parameter conditional on the current values of all the other parameters, and then sampling a random value from the posterior distribution. This procedure can be shown to converge eventually on the posterior distributions of the parameters. In the current study, diffuse uniform prior distributions (from 0 to 100) were used for all parameters, and the residual variance was assigned a weakly informative uniform prior (from 0.01 to 100) [42]. We ran two chains for 10000 iterations after a burn-in of 5000 iterations and thinned the chains by retaining every 10^th^ value, so that the resultant posterior distributions contained 1000 values each. In other words, for each of the four lesion (pathogen) datasets, we obtained 1000 vectors of fitted parameter values and associated residual variance. We assessed model convergence (on the same posterior distributions) using the Brooks-Gelman-Rubin diagnostic [43]. A value close to 1 indicates convergence of parallel chains: the diagnostic was < 1.1 for all model parameters for the Pba, Pbr, Ppa, and Dsol datasets.

The deviance information criterion (DIC) was used as a crude index to compare models [44]. The DIC is the sum of the mean deviance [deviance = −2 log (likelihood)] and of the effective number of parameters (pD; the posterior mean of the deviance minus the deviance of the posterior means). A difference of more than 10 in DIC was taken as a rough index of difference between two models and ruled out the model with the higher DIC [44]. When the difference in DIC was less than 10, we selected the model with the best goodness of fit, i.e., with the lower deviance. To quantify the accuracy of the mean estimated parameter values, the goodness of fit of the selected model to the observed data for each pathogen was assessed using the root mean square error. The quality or predictive ability of the selected model was assessed using a Bayesian ‘p-value’ analogue calculated from the predictive posterior distribution [45]. The Bayesian p-value quantifies the proportion of times when the lack of fit of a perfect data set (a replicated data set generated using the same model that is fitted to the actual data set) is greater than the lack of fit of the actual data set. A Bayesian p-value close to 0.5 indicates that the model is not consistently under-predicting (*p*-value near 0) or over-predicting (*p*-value near 1) [46]. Bayesian p-values of <0.1 and >0.9 were considered extreme values and, hence, indications of where the predictive ability of the model was poor.

### Weather data for climate change scenarios

Raw monthly 25 km gridded values of mean temperature from the UK Met Office Climate Projections database (UKCP09) 11-member ensemble of spatially coherent climate projections (SCPs) were used to drive the disease severity model. The SCPs provide the best estimates for modelling and summarizing the potential effects of future climates on crop disease epidemics in the UK as they are fully coherent across different locations, allowing the user to consider climate change at more than one location in a way that captures the relationship between the different locations, and to average projections across user defined land areas. They include both internal modelling variability (using perturbed physics ensembles) and external modelling variability (from the use of different General Circulation Models, or GCMs), as well as information on climate variability. The SCPs provide 11 equally plausible snapshots of climate change for a number of different emissions scenarios and time periods and are freely available for download at http://ukclimateprojections.defra.gov.uk/. Three, thirty-year future time-slices (centred on the 2040s, 2060s, and 2080s) and three climate forcing scenarios (UKCP09 Low, Medium and High CO_2_ emissions) were used. UKCP09 1961–1991 baseline data were used for comparison, making a total of ten climate change scenarios. Monthly temperature values from June to September were used, as these are the months during which *Pectobacterium* and *Dickeya* species are prevalent in GB.

**Fig 1:**
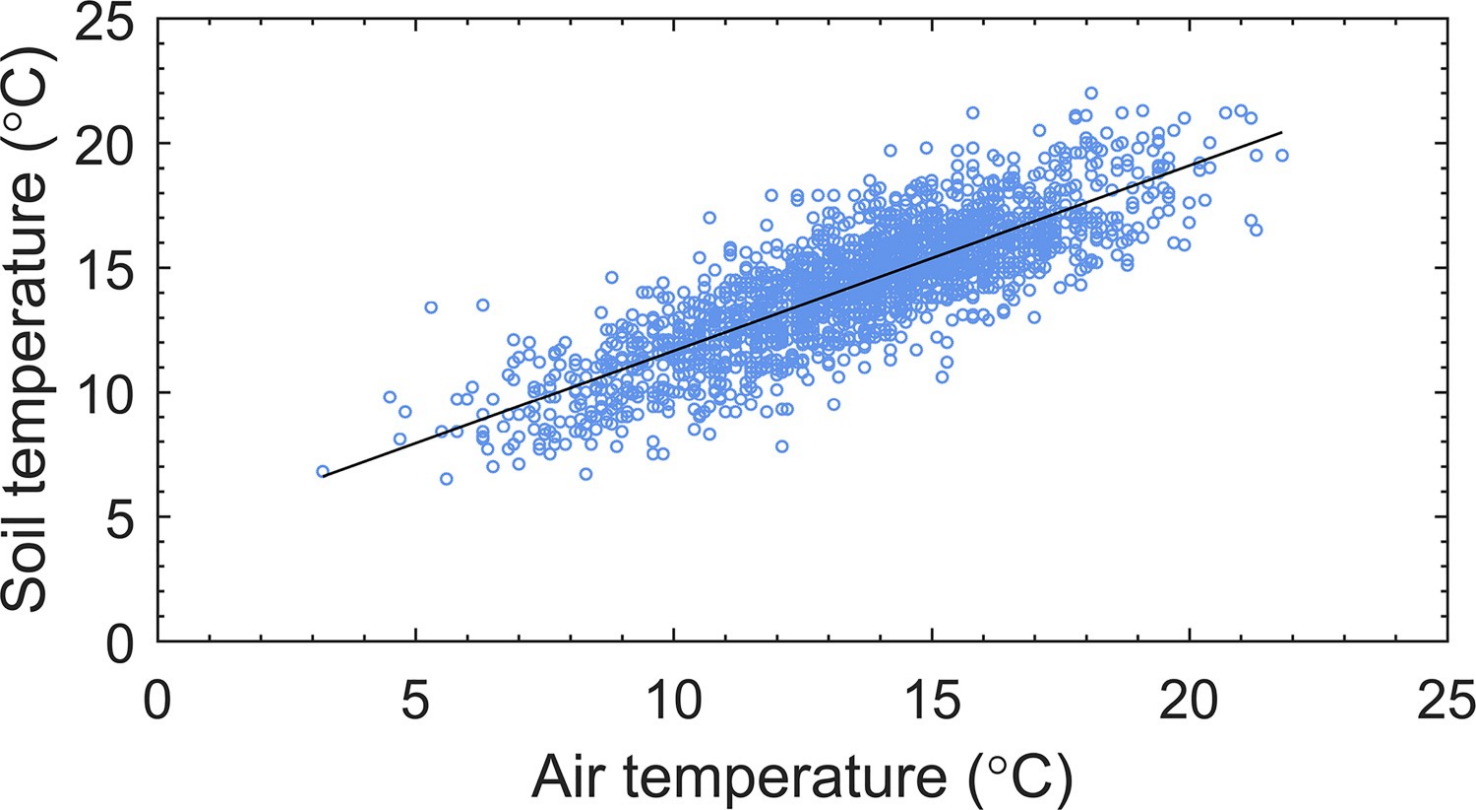
Relationship between mean daily soil (20 cm depth) and air temperatures. Linear regression, *y* = 4.22 + 0.74 *x*, R^2^ = 0.68.

**Fig 2:**
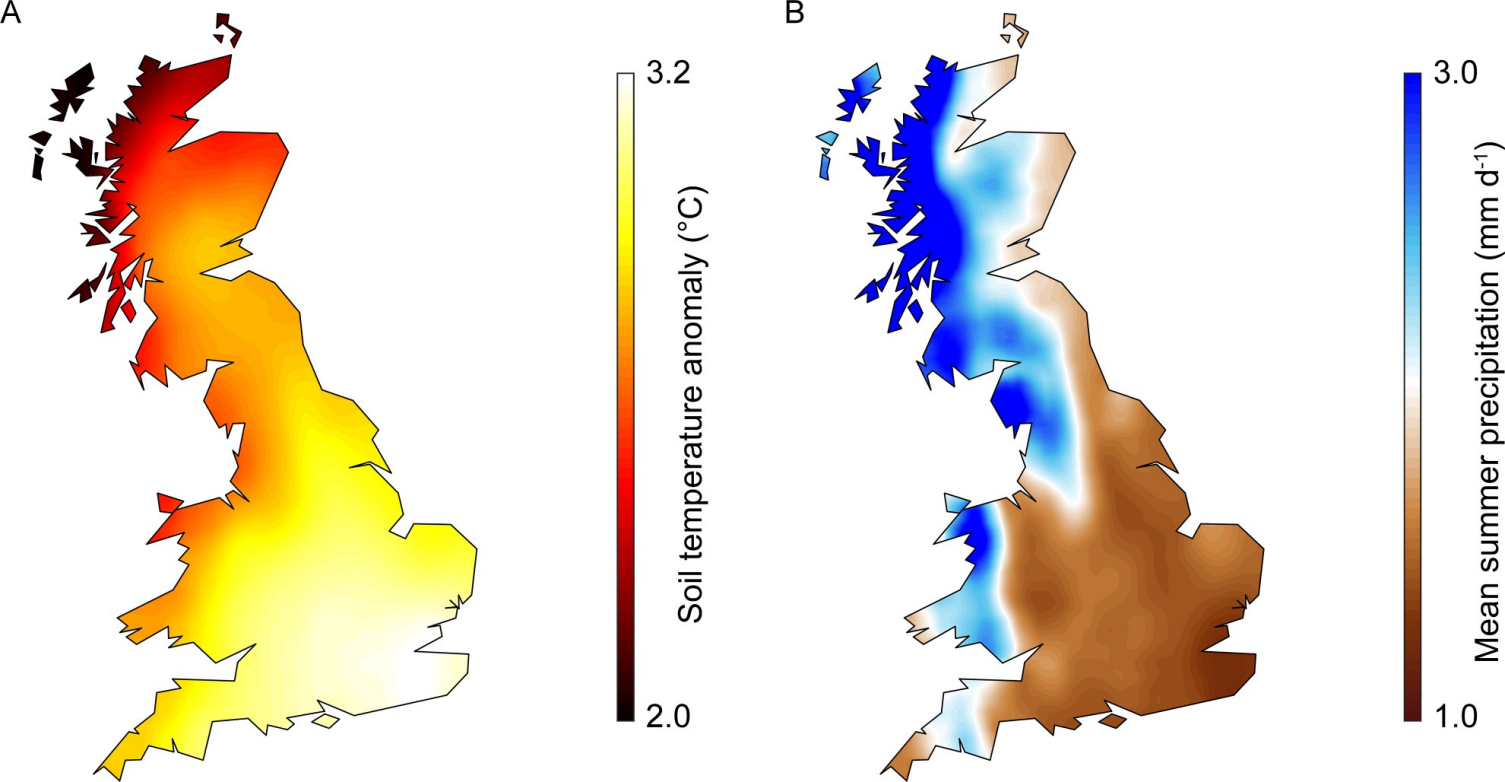
Example maps of climate change projections. (A) Difference in soil temperature compared to current baseline values, calculated using the 1^st^ member of the UKCP09 SCP ensemble of air temperature changes for June under a high CO_2_ emissions scenario in the 2080s. (B) Summer mean precipitation rate for the same scenario ensemble member.

We used the relationship between mean daily air (1.25 m) and soil temperatures (20 cm depth), derived from a 15-year time series (1999–2014) of data from Mylnefield weather station (56°27'25.04"N, 3°4'23.77"W), to convert projected seasonal air temperatures to future seasonal soil temperatures (Fig 1). Although this empirical relationship may differ among potato production areas in GB, it provides a reasonable approximation for the purposes of a first climate change risk assessment for non-indigenous *Pectobacterium* and *Dickeya* species in GB (Fig 2A).

UKCP09 SCP data for summer (June-August) mean precipitation (mm d^-1^) were used to identify potentially suitable locations for rainfed potato production in Scotland, England and Wales under future climates (Fig 2B)

### Crop distribution data

**Fig 3:**
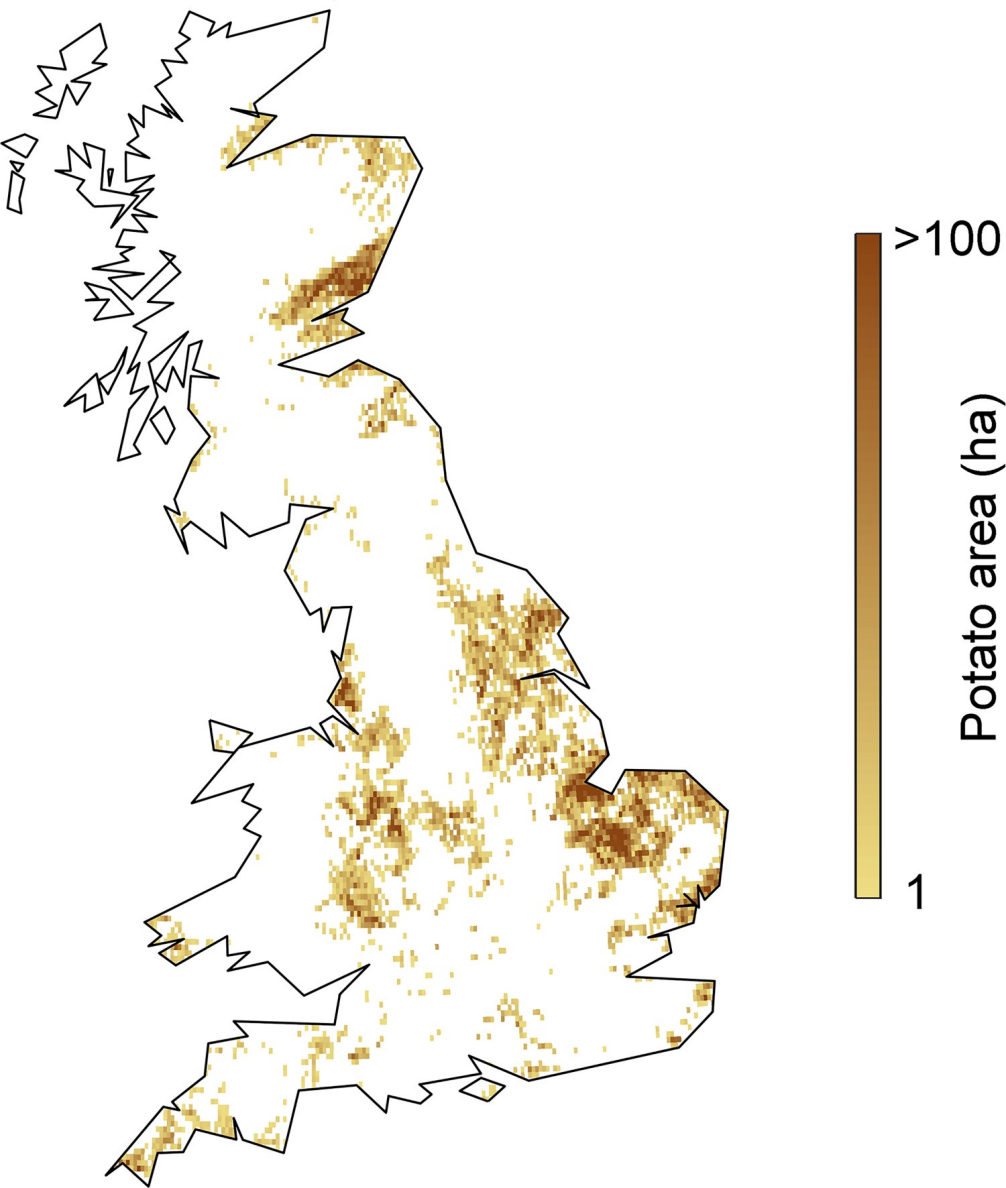
Spatial coverage of potato crops in Great Britain in 2015. Data have been aggregated to a 2.5 arcminute (approximately 5 × 5 km grid) for illustrative and confidentiality purposes.

We initially proceed under the assumption that the current spatial coverage of potato crops provides a reasonable proxy indicator of future coverage (this assumption is later relaxed in the adaptation scenarios). Polygon data defining the spatial coverage of potato crops in 2015 (19,523 crops) were provided by AHDB Potatoes (http://potatoes.ahdb.org.uk/image/main-production-areas) (Fig 3). These data are protected and are only available to the public in a high level summary form using the link provided. The data were rasterized to a 25 km binary grid (containing potato and non-potato) matching the resolution of the climate change data.

### Projected change in aggressiveness in tubers

Monthly values for disease severity (lesion diameter) in tubers were generated for the four *Pectobacterium* and *Dickeya* species using the 11-member ensemble of UKCP09 SCPs for all ten of the CO_2_ emissions scenarios for each grid cell containing potato. For each projected value of disease severity (Eq 1) we then drew 100 values of residual error, where the residual variance is different for each parameter vector. This gave a super-ensemble of 1.1 × 10^6^ (1000 parameter vectors × 100 values of residual error × 11 SCPs) disease severity values for each combination of pathogen, potato grid cell location, month, and CO_2_ emissions scenario. We investigated the effects of geographic variability in climate on future aggressiveness in tubers by grouping projected values within the three constituent countries of Great Britain (Scotland, England, and Wales). Results for future climates were taken relative to those for baseline conditions and presented as percentage changes to current aggressiveness in tubers using boxplots for each of the four pathogens: i.e., relative change = (severity_future_−severity_current_) / severity_current_ × 100. Results are summarized using boxplots (computed from the super-ensemble of projected values) to quantify uncertainty in predicting climate, uncertainty in the model parameter values, and uncertainty due to model residual error. This gave a total of 48 boxplots per CO_2_ emissions scenario: 4 pathogens × 4 months × 3 countries.

### Projected relative differences in aggressiveness

Projected lesion diameters for Pbr, Ppa, and Dsol (the main causative agents in Europe) were also directly compared to the corresponding values for Pba (the GB strain) in order to investigate the potential for widespread establishment of the European strains and species in GB, on the basis of their differential capacity to cause disease in tubers under future climates. All lesion diameters were normalized by the maximum projected Pba lesion diameter, and then displayed using radar plots to visualize the aggressiveness of the European strains and species relative to Pba.

### Adaptation scenarios–Optimized crop locations

Extreme climate change will almost certainly require relocation of potato production from the semi-arid east to rain-fed production in the west, with all the necessary capital investments in farming businesses and regional infrastructure [30, 31]. To investigate the impact of such a shift on the aggressiveness of *Pectobacterium* and *Dickeya* spp. in tubers, we reassigned the potato-containing cells in Scotland, England and Wales, respectively, to the (land) grid cells with the highest summer (June to August) mean precipitation rate under each climate change scenario. In other words, we ‘moved’ the potato crops in each country (typically on the semi-arid eastern seaboard; Fig 3) to the wettest locations in that country (typically more westerly locations; Fig 2B). In reality, the determinants of land suitability for potato production are complicated by a large number of climate, soil, water abstraction and other crop management factors, but we proceed under the simplifying assumption that movement to areas of higher rainfall will be a key driving factor in the future, for the purposes of simulation. We repeated all climate change projections, and results for future climates were taken relative to those for baseline conditions and presented as percentage changes to current aggressiveness in tubers for each of the four pathogens: i.e., relative change = (severity_future_−severity_current_) / severity_current_ × 100. Results are summarized using stem plots to compare median changes to severity in current (actual) potato crop locations and median changes to severity in optimized crop locations. This gave a total of 96 data values per CO_2_ emissions scenario: 4 pathogens × 4 months × 3 countries × 2 crop distributions.

## Results

### Tuber assay and model fit

**Fig 4:**
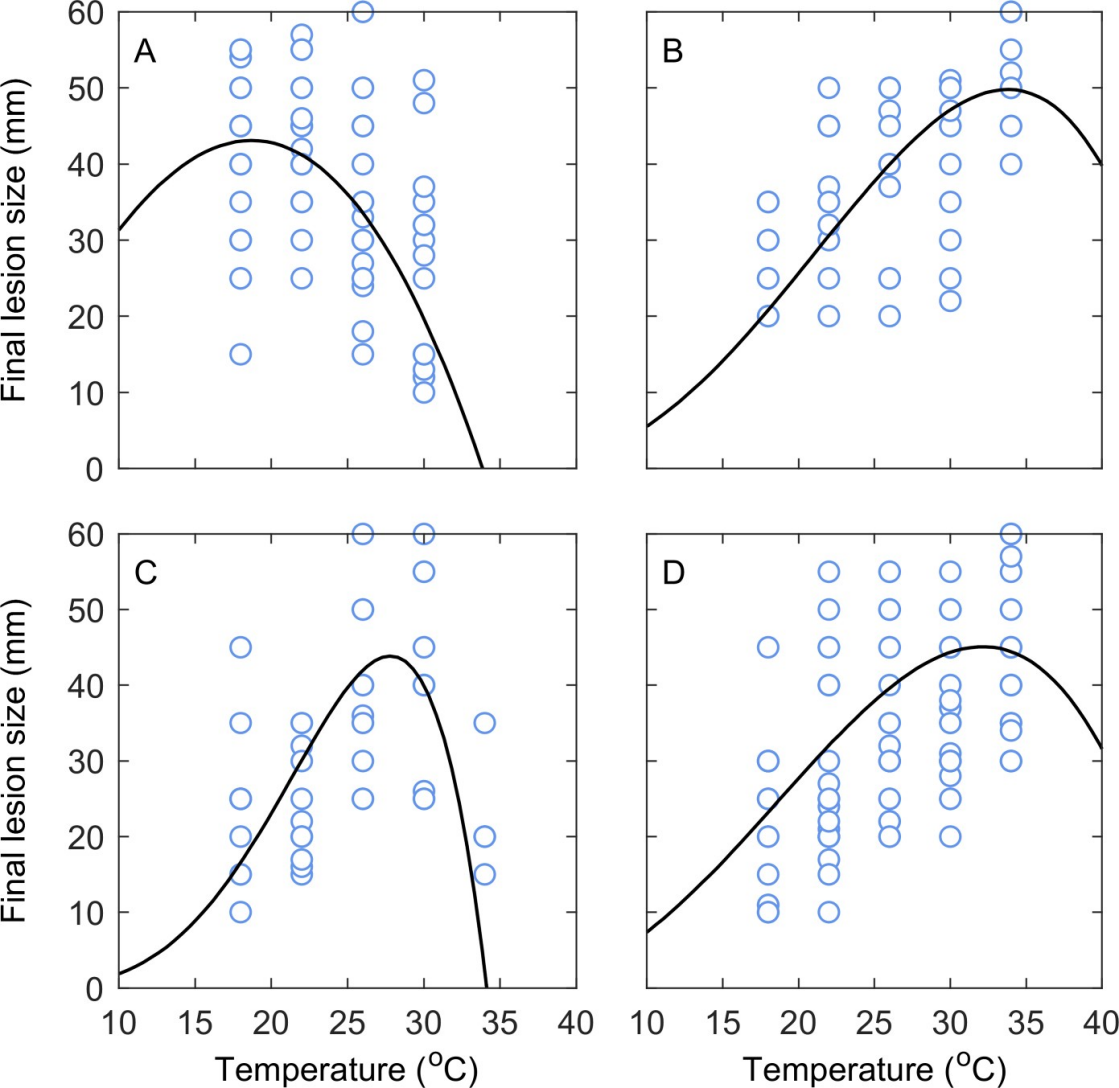
Effect of temperature on lesion size of *Pectobacterium* and *Dickeya* spp. (A) *P*. *atrosepticum*, (B) *P*. *carotovorum* subsp. *brasiliense*, (C) *P*. *parmentieri*, and (D) *D*. *solani*. Data markers are observed values and solid lines show predicted values from a Beta function (Eq 1).

There were clear differences in temperature response among species and strains (Fig 4); raw data can be found in the supplementary S1 Table. The response was clearly unimodal for Pba (Fig 4A) and Ppa (Fig 4B), while the division between sub-optimum and supra-optimum temperatures was less apparent for Pbr (Fig 4B) and Dsol (Fig 4D). The difference in DIC between the linear model and the beta function (Eq 1) was > 10 for Pba and Ppa, and DIC and pD values were lower for the beta function for Pbr and Dsol, therefore the beta function corresponded to the best model that could be obtained basing our choice on the DIC criterion. The overall fit of the beta function was satisfactory with a RMSE (root mean square error) of 8.32, 7.50, 6.92 and 8.72 mm for the Pba, Pbr, Ppa, and Dsol data, respectively. We obtained Bayesian p-values of 0.54, 0.53, 0.54, and 0.53 for the beta function for Pba, Pbr, Ppa, and Dsol data, respectively, indicating an adequate model fit with no tendency for under- or over-prediction. The mean estimated parameter values (Eq 1) were: *L*_max_ = 44.12 mm, *T*_max_ = 34.03°C, *T*_opt_ = 17.84°C for *P*. *atrosepticum*; *L*_max_ = 48.12 mm, *T*_max_ = 47.16°C, *T*_opt_ = 32.62°C for *P*. *carotovorum* subsp. *brasiliense*; *L*_max_ = 40.36 mm, *T*_max_ = 35.1°C, *T*_opt_ = 27.51°C for *P*. *parmentieri*, and *L*_max_ = 43.3 mm, *T*_max_ = 47.1°C, *T*_opt_ = 33.33°C for *D*. *solani*.

### Projected change in aggressiveness in tubers

**Fig 5:**
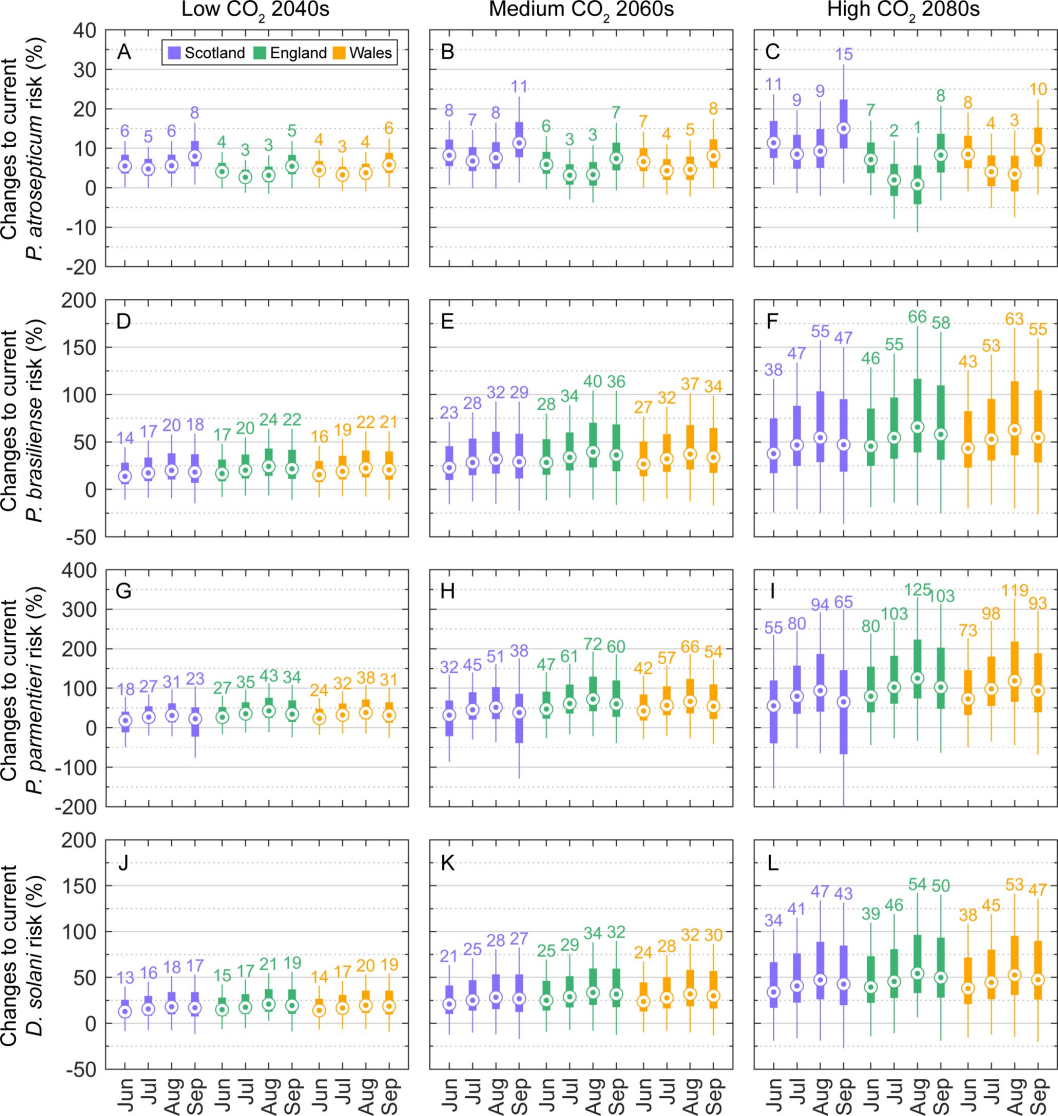
Projected change in disease severity (lesion size) caused by *Pectobacterium* and *Dickeya* species in GB, for three CO_2_ emissions scenarios. (A-C) *P*. *atrosepticum*, (D-F) *P*. *carotovorum* subsp. *brasiliense*, (G-I) *P*. *parmentieri*, and (J-L) *D*. *solani*. Results for different CO_2_ emissions scenarios are given in columns. Projected values are expressed relative to the 1961–1991 baseline (current) climatology to show the proportional response: (severity_future_−severity_current_) / severity_current_ × 100. Boxplots represent uncertainty due to climate prediction, parameter estimation, and model residual error within the three constituent countries of Great Britain. Boxes extend from first to third quartile, medians are marked in each box and labelled above the upper whiskers for clarity, and whiskers extend to the 5^th^ and 95^th^ percentiles.

Projections suggest that as the weather in the UK changes, the aggressiveness of Pba in tubers will increase (Fig 5A–5C). Results are presented for the low CO_2_ emissions scenario in the 2040s, the medium CO_2_ emissions scenario in the 2060s, and the high CO_2_ scenario in the 2080s, as the other scenarios produced intermediate results. Pba severity increased consistently with time (decades) and CO_2_ emissions, with smaller increases projected for July and August, as both baseline and projected temperatures were nearer the optimum values for Pba lesion development during the warmer summer months (Fig 4). There was limited geographic variability in the response of Pba to climate change: the mean difference in median monthly changes to current Pba severity among the constituent countries of GB was 1.4, 2.4, and 4.4% for the low CO_2_ 2040s, medium CO_2_ 2060s, and high CO_2_ 2080s scenarios, respectively.

Pbr aggressiveness in tubers also increased consistently with time and CO_2_ emissions, but unlike Pba the percentage changes to current severity decreased in September (Fig 5D–5F). This is due to the initial curvilinear or convex portion of the Pbr temperature-response curve; baseline and projected air temperatures for September corresponded to a less steeply ascending portion of the Ppa response curve than August temperatures (Fig 4B). The percentage changes to current Pbr severity were notably higher than those for Pba. This should not be interpreted as implying a competitive advantage over Pba, but rather that temperatures are expected to become relatively (compared to current conditions) more favourable for Pbr development in tubers than Pba. Geographic variability in the response of Pbr to climate change was negligible, with a mean difference in median monthly changes to current Pbr severity among the constituent countries of GB of 2.3, 4.3, and 6.3% for the low CO_2_ 2040s, medium CO_2_ 2060s, and high CO_2_ 2080s scenarios, respectively.

Percentage changes to the aggressiveness of Ppa in tubers also increased consistently with time and CO_2_ emissions, and similar to Pbr, smaller changes were projected for September than August (Fig 5G–5I). The percentage changes to current severity were notably the highest of the four pathogens investigated. Again, this does not imply a competitive advantage, but suggests potential for Ppa to become a higher consequence pathogen in potato crops in GB than present. There was notable geographic variability in the response of Ppa to climate change, with a mean difference in median monthly changes to current Ppa severity among the constituent countries of GB of 6.8, 12.3, and 19.3% for the low CO_2_ 2040s, medium CO_2_ 2060s, and high CO_2_ 2080s scenarios, respectively.

Changes to current Dsol severity (Fig 5J–5L) were comparable to those for Pbr under all emissions scenarios, although slightly lower. This is due to the similarity of their temperature-response curves (Fig 4D). Geographic variability in the response of Dsol to climate change was also comparable to Pbr, with a mean difference in median monthly changes to current Dsol severity among the constituent countries of GB of 1.6, 3.0, and 4.1% for the low CO_2_ 2040s, medium CO_2_ 2060s, and high CO_2_ 2080s scenarios, respectively.

### Projected relative differences in aggressiveness

**Fig 6:**
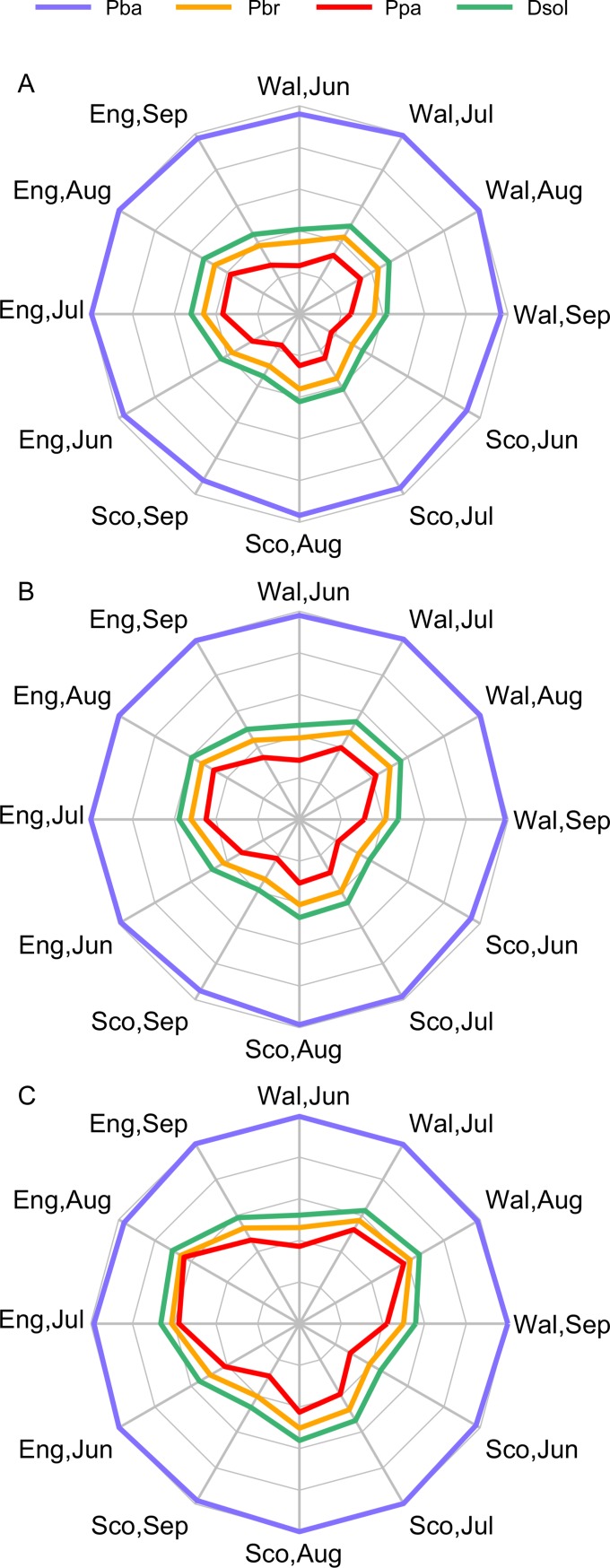
Radar plot showing projected change in aggressiveness (mean lesion size) of three *Pectobacterium* and *Dickeya* species relative to *P*. *atrosepticum* under three CO_2_ emissions scenarios. (A) low CO_2_ emissions 2040s, (B) medium CO_2_ emissions 2060s, and (C) high CO_2_ emissions 2080s. Data are normalized by the maximum projected *P*. *atrosepticum* lesion size. Pba = *P*. *atrosepticum*, Pbr = *P*. *carotovorum* subsp. *brasiliense*, Ppa = *P*. *parmentieri*, and Dsol = *D*. *solani*.

Results are presented for the low CO_2_ emissions scenario in the 2040s, the medium CO_2_ emissions scenario in the 2060s, and the high CO_2_ scenario in the 2080s, as the other scenarios produced intermediate results. Pba was able to produce consistently larger lesions under all climate change scenarios, followed by Dsol, Pbr, and Ppa (Fig 6). However, as projected soil temperatures increased through the climate change scenarios, the difference in aggressiveness between Pba and the non-indigenous European strains and species decreased. This was most evident in England and Wales in July and August.

### Adaptation scenarios–Optimized crop locations

**Fig 7:**
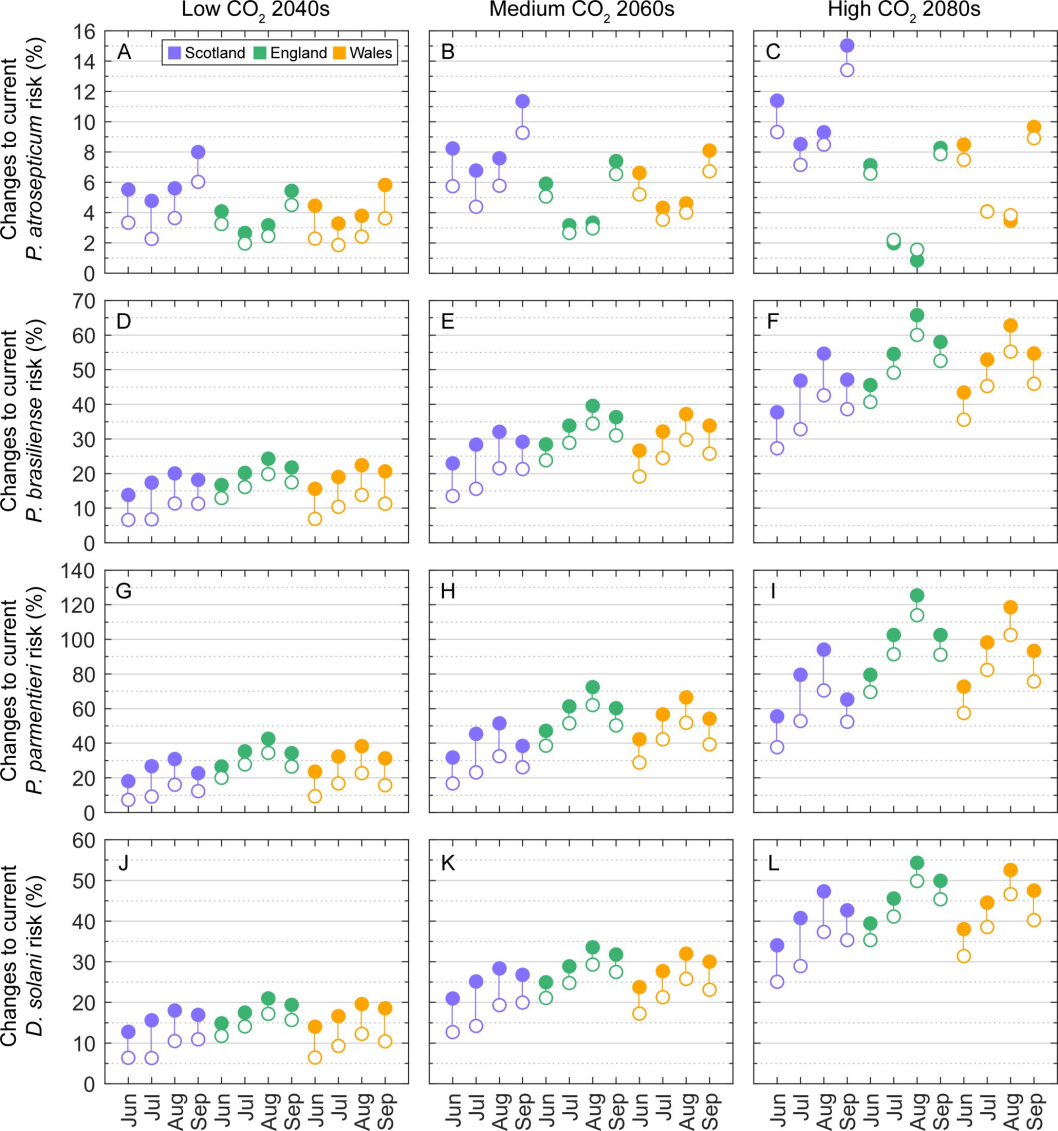
Stem plot of projected change in disease severity (lesion size) caused by *Pectobacterium* and *Dickeya* species in GB after shifting crop production to the areas of highest summer rainfall in each constituent country. (A-C) *P*. *atrosepticum*, (D-F) *P*. *carotovorum* subsp. *brasiliense*, (G-I) *P*. *parmentieri*, and (J-L) *D*. *solani*. Results for different CO_2_ emissions scenarios are given in columns. Projected values are expressed relative to the 1961–1991 baseline (current) climatology to show the proportional response: (severity_future_−severity_current_) / severity_current_ × 100. Solid data markers show median projected changes to current severity in actual potato crop locations (Fig 5) and empty data markers show median projected changes to current severity in optimized crop locations.

Moving potato locations to the areas of highest rainfall in the country served to reduce the projected median (of all months and countries of GB combined) change to current Pba severity from 4.6 to 3.0%, 6.4 to 5.0%, and 7.4 to 6.7% under the low CO_2_ 2040s, medium CO_2_ 2060s, and high CO_2_ 2080s scenarios, respectively (Fig 7). The projected median (of all months and countries of GB combined) change to current Pbr severity decreased from 19.1 to 12.0%, 31.6 to 23.9%, and 51.9 to 43.5% under the low CO_2_ 2040s, medium CO_2_ 2060s, and high CO_2_ 2080s scenarios, respectively. The projected median (of all months and countries of GB combined) change to Ppa severity decreased from 30.2 to 17.8%, 52.2 to 37.9%, and 90.6 to 74.0% under the low CO_2_ 2040s, medium CO_2_ 2060s, and high CO_2_ 2080s scenarios, respectively (Fig 4). The projected median (of all months and countries of GB combined) change to Dsol severity decreased from 17.0 to 11.0%, 27.8 to 21.1%, and 44.6 to 37.8% under the low CO_2_ 2040s, medium CO_2_ 2060s, and high CO_2_ 2080s scenarios, respectively (Fig 7). The projected median increases to current severity (all months, species, and CO_2_ emissions scenarios combined) were reduced by a factor of 1.6, 1.3, and 1.4 in Scotland, England and Wales, respectively.

## Discussion

In this study we developed a parsimonious but biologically plausible model to describe the effects of temperature on disease severity (lesion size) of GB and European *Pectobacterium* and *Dickeya* spp. in potato tubers, using a Bayesian framework and data from controlled environment experiments. This was combined with probabilistic climate change data and potato crop distribution data to project the differential capacity to cause disease in tubers under future climates in GB production areas, and provide a complete characterisation of model uncertainty and uncertainty in predicting climate. A key finding for the GB potato industry is that the greatest future increases in aggressiveness in tubers for Pbr and Dsol, the predominant blackleg (symptoms on stems as opposed to tubers) and soft rot strains in Europe, are projected to occur late in the season in August and September. Our first take-home message to the GB potato industry is that this finding reinforces the need to maintain current best practice for managing potato blackleg in GB: to kill haulm in crops well in advance of harvest and to harvest crops as early as possible [47], as current Pba incidence and severity are also significantly higher in GB in August and September. A second key finding for the GB potato industry is that temperatures during July and August in England and Wales are currently near the optimum for Pba, the predominant blackleg strain in GB. The projected changes to current Pba severity during July and August were therefore small and declined with increasing time and level of CO_2_ emissions (Fig 5). Where temperature increases exceed the pathogen’s thermal optimum, we might see the emergence of a bimodal pattern of disease, where Pba severity levels peak both early and late in the season but decrease during the midsummer. The potential for an increase in Pba early in the season could be managed by having pectolytic bacterial levels assessed on seed prior to planting. Although diagnostics are only indicative of levels on tubers at the time of testing, it would still provide an indication of potential disease development. Thus, our second take-home message for the GB potato industry would be to consider increased diagnostic testing on seed prior to planting in the future.

For a number of years the GB potato industry has been concerned about the potential establishment of the predominant European *Pectobacterium* and *Dickeya* species and strains, therefore this issue warrants further consideration. We found that the mean projected soil temperatures across all potato crop locations in GB were 15.0, 17.1, 17.3, and 15.1°C for June through to September under the low CO_2_ emissions 2040s scenario, and 18.0, 20.7, 21.6, and 18.7°C for June through to September under the high CO_2_ emissions 2080s scenario. As is evident from the tuber slice assay (Fig 4), these temperatures are all close to the optimal value for Pba lesion development and highly sub-optimal for the predominant European blackleg and soft rot pathogens (Pbr, Ppa, and Dsol). This provides evidence that temperatures are a limiting factor for the widespread establishment of these pathogens in GB. Nevertheless, in the tuber slice assay there were still significant levels of disease caused by Pbr, Ppa, and Dsol at the lower temperatures. The experimental results therefore show that the predominant European pathogens can cause disease under conditions currently seen in GB, but are not expected to outcompete Pba and become the predominant blackleg and soft rot pathogens in the future, at least based on their projected capacity to cause disease in tubers under future GB conditions (Fig 6). These findings do not preclude, however, the potential for the European pathogens to gain a foothold in England and Wales, given the expected increase in the suitability of temperature conditions for these species. Both Ppa and Pbr have been confirmed in GB; an annual blackleg survey conducted in England and Wales identified Ppa and Pbr as the causes of blackleg in 7.3% and 0.8% of stocks in which the disease was found during official inspections in 2015 [6]. Nevertheless, there is no evidence that the incidence of disease caused by strains of pectolytic bacteria other than Pba has increased over the last 6 seasons [6]. All crops in which Pbr has been found have been grown directly from seed imported from the Netherlands, with the exception of single cases in 2014 and 2015, both in seed from German origin. Ppa has also been found to cause disease in crops grown from seed of Danish, German and Netherlands as well as GB origins. The widespread origins of seed stocks infected with Pbr and Ppa suggest that both pathogens have either spread rapidly or have been circulating in European potato for many years. Dsol has also been confirmed in GB; it was first isolated on a seed potato crop (non GB origin) in England and Wales in 2007 [48], and in Scotland was found in two ware crops of non-Scottish origin in 2009 [49]. Results from the annual surveys strongly suggested that the principal source of Dsol was infected seed of non-UK origin [6]. In all but one finding of disease caused by Dsol the planted seed stock had either been directly imported from the Netherlands or previously multiplied in England and Wales from a seed stock originating in the Netherlands. It was therefore concluded that the source of *Dickeya* infecting potatoes in England and Wales was exclusively seed of non-UK origin and that there was no evidence for horizontal spread of *Dickeya* spp. to seed stocks of UK origin. The lack of observed horizontal spread could partly be explained by the lower aggressiveness of Dsol relative to Pba under GB conditions that was highlighted in this study. Scotland implemented an extensive monitoring scheme that included growing crop surveys and post-harvest tuber testing of all non-indigenous origin seed and ware crops, and other high risk crops, such as irrigated stocks, stocks grown near watercourses contaminated or previously contaminated with *Dickeya* spp., and close contact crops (grown on farms with previous Dickeya positives). Scotland also introduced new legislation in 2010 that prescribes ‘nil tolerance’ for symptoms caused by Dickeya spp. in all classes of seed potatoes. Since the introduction of this new legislation, no new *Dickeya* infections have been found [50]. There has also been a marked decline in the number of non-Scottish origin crops grown in the country, due to use by the industry of Scottish-origin seed. Thus, our third take-home message for the GB potato industry is to reinforce the importance of the current high standards of *Dickeya* monitoring as the weather in Britain warms, to prevent this pathogen gaining a foothold in potato growing areas. In addition, the ‘nil tolerance’ approach enforced by Scotland could ultimately be beneficial in England and Wales.

Another finding of interest for the potato industry is the ramifications of climate-change induced shifts in agricultural planting patterns. The influence of temperature (and thus crop location) on aggressiveness in tubers suggests potential for the design of disease suppressive landscapes, where a more strategic approach to the introduction of new potato production areas is adopted. In this study we investigated a shift in GB potato production from the semi-arid east to rain-fed production in the west, and its impact in mitigating future increase in aggressiveness in tubers. This adaptation strategy was most effective in Scotland, as Scotland has the coolest current and projected weather in GB, and least effective in England, which has the warmest temperatures and the most widespread distribution of potato crops. It is likely this adaptation strategy would have been much more effective in general had we used the polygon data defining the spatial coverage of potato crops as opposed to rasterizing the data to a coarse 25 km binary grid (giving less options for crop relocation), or had we accounted for the area of crop within each grid cell and modelled the area affected by disease over time. However, both these options would have been prohibitively computer-intensive given the Bayesian parameter estimation method used in this study, where over 1 million disease severity values were generated for each combination of pathogen, potato grid cell location, month, and CO_2_ emissions scenario.

Our synthesis of crop distribution and probabilistic climate change data, together with the application of Bayesian parameter estimation to a novel model describing the effects temperature on aggressiveness in tubers, enabled the development of a super-ensemble based probabilistic projection approach that accounted systematically for the uncertainties not only from the emission and climate change scenarios, but also from the biophysical processes in the model. This work highlights the importance of spatial context in climate change risk assessments for crop pathogens, as the efficacy of adaptation strategies varied between the constituent countries of GB. We were able to demonstrate the utility of our approach in providing useful information to guide potato industry management practices and government strategies for agricultural adaptation to climate change. Although the aggressiveness of Pba is projected to increase slightly in GB in the future, and results indicate a modest potential for the predominant European *Pectobacterium* and *Dickeya* spp. to gain a foothold, the GB potato industry is well positioned to continue to thrive via current best management practices and continued reinforcement of existing legislation. The methodology developed in this study is extendable to other potato blackleg and soft rot pathogens and can be applied using standard GCM output.

## Supporting information

S1 Table Data from tuber slice assay. (CSV)
